# Systematic Review of Antifungal-Induced Acute Liver Failure

**DOI:** 10.7759/cureus.18940

**Published:** 2021-10-21

**Authors:** Eyad Gadour, Ahmed Kotb

**Affiliations:** 1 Gastroenterology and Hepatology, University Hospitals of Morecambe Bay National Health Service (NHS) Foundation Trust, Lancaster, GBR; 2 Hepatobiliary Surgery, Glan Clwyd Hospital, Wales, GBR

**Keywords:** drug induced liver injury, fluconazole, hepatotoxicity, antifungals, acute liver failure

## Abstract

Antifungals are effective antimicrobial agents broadly used in medical practice. Severe acute liver failure from oral or IV administration of antifungals is a rare but long-standing clinical challenge. We aimed to approximate the risk of clinical acute liver injury among users of oral antifungals in the general population. This review was completed based on the Preferred Reporting Items for Systematic Reviews and Meta-Analyses (PRISMA) guidelines.

Six articles were included, comprising case reports and cohort studies, after eliminating duplicate publications. No randomized control studies were found. In all studies, the duration of antifungal use was associated with significantly increased liver enzyme levels.

Although it is not very common for patients on antifungals to develop acute liver failure, the prognosis is often good with swift discontinuation of the drug and proper treatment. Liver function evaluation before treatment and periodic monitoring every three to six weeks after commencement of treatment is suggested.

## Introduction and background

Oral antifungals have been implicated in many case reports of hepatotoxicity and serious liver injuries in the last few decades [1]. Antifungal-induced hepatic injury is often characterized as an acute, cholestatic, or mixed hepatocellular-cholestatic response [1]. The reaction generally resolves with discontinuation of treatment, but some liver damage can be chronic. Serious hepatic side effects of oral antifungal agents are considered rare, but reported incidence rates vary widely and depend on the agent [2].

Fungal infections are a leading cause of ailment and death in immunocompromised and very ill patients [3]. Although antifungal drug options have increased in recent years, yet effective management significantly depends on the early and proper treatment that improves efficacy and safety [4]. Existing liver disease can be a contraindication for antifungal administration due to safety concerns. The liver is a major component of drug metabolism, and hepatic disease can dramatically alter the pharmacokinetics of antifungal drugs [5] due to impaired clearance, liver blood flow, biliary excretion, and plasma protein binding. Such patients are less likely to tolerate drug-induced liver injury (DILI) than healthy people [6]. In addition, patients with cirrhosis are more prone to drug-related side effects, such as kidney failure, intestinal bleeding, and hepatic encephalopathy [7]. A hepatic function can influence drug-drug interactions (DDI) due to reduced drug uptake and inhibition of metabolizing enzymes [8].

Hepatotoxicity is defined as chemical-induced liver damage [9]. There is a known link between ketoconazole and hepatotoxicity [10]. However, the early evidence suggested that ketoconazole-induced hepatotoxicity was mild, rarely fatal, and could be reversed upon drug discontinuation. In the United Kingdom, hepatotoxicity was reported in one of 15,000 patients in the first decade after oral ketoconazole market approval [11].

DILI is one of the leading causes of acute and chronic liver diseases. To diagnose this condition, it is essential to distinguish detected biochemical abnormalities from actual hepatic dysfunction. DILI is typically characterized by increased levels of hepatic enzymes resulting from the effects of an active drug or its metabolites on the liver [12]. This biochemical imbalance is not necessarily associated with clinically significant liver dysfunction because the liver has significant healing properties [13]. However, DILI may cause hepatic dysfunction, which manifests as hyperbilirubinemia and coagulopathy, or severe liver failure, presenting with jaundice and hepatic encephalopathy [14]. Most drugs work by interfering with one or more cellular/molecular activities; they, therefore, have the potential to produce less desirable reactions [15].

The antifungals fluconazole and itraconazole are considered relatively safe; they have been associated with only minor changes in liver function tests that usually do not require interruption of treatment. Fluconazole is widely used in the treatment of various fungal infections. It is found in oral and parenteral forms and is different from other azoles as it is primarily metabolized by the kidneys rather than the liver [16]. The mechanisms by which it causes temporary elevation of transaminases are unknown, although idiosyncratic reactions appear to be involved. There have been two reported cases of marked liver toxicity due to fluconazole and itraconazole requiring the suspension of these azoles; in these cases, the symptoms resolved immediately after drug withdrawal [17].

Ketoconazole has a higher incidence of hepatic damage than other systemic antifungals [18]. In a randomized clinical trial, patients with ketoconazole-treated onychomycosis were three times more likely to develop hepatitis than patients treated with griseofulvin [19].

Cases of liver damage have also been reported in conjunction with griseofulvin [20], itraconazole [21], and terbinafine [22], and several fluconazole-related cases have occurred in severely immune-depressed patients [23]. In a recent post-marketing study of 25,884 patients treated with terbinafine, two cases of symptomatic hepatic injury related to antifungal treatment were identified [24]. The risk of liver damage and hepatic dysfunction caused by an antifungal agent depends on several factors: the chemical characteristics of the agent, genetic predisposition, comorbidities including primary hepatic disease, associated hepatotoxic drugs and DDIs, and fungal infection severity and involvement of the liver [25]. Thus, the mechanisms of DILI can be multi-factorial.

Chronic liver disease patients are at increased risk of developing diseases that lead to life-threatening conditions, such as sepsis and hepatic encephalopathy [26], for several reasons, including dysfunctional immune response, increased intestinal permeability resulting in changes in the quantity and quality of gut microorganisms, and genetic predisposition, which adds to the transmission of fungal infections from the gut to the circulatory system [27]. The onset of fungal infections has been linked to the emergence of many complications, such as severe kidney damage and multiple organ failure, all of which result in short- or long-term mortality [28]. Fernandez et al. found that 2% of hospitalized patients with acute-on-chronic liver disease (ACLD) had fungal infections [29]. At any stage of liver disease, either compensated or non-compensated, and including severe or chronic liver failure, the added complication of fungal infection leads to an increased risk of death.

Overall, there is no clear consensus in the published literature about the use of antifungal agents in patients with pre-existing liver disease. The understanding of liver damage caused by antifungal drugs in patients with hepatic impairment is not clear, and recommendations for dose adjustment in these cases are not straightforward. Most information about antifungal regimens dosing comes from clinical trials and pharmacokinetic studies which included only a few patients with varying degrees of liver disease. The manufacturers of some antifungals recommend dose reduction in cases of hepatic dysfunction, while others do not [30].

We aimed to summarize the current data on the pharmacokinetics of antifungals for these individuals and to increase clinical awareness of the proper use of various antifungal compounds in the case of liver injury.

Materials and methods

This research was completed based on the Preferred Reporting Items for Systematic Reviews and Meta-Analyses (PRISMA) guidelines to ensure transparency, completeness, and robustness of reporting.

Inclusion and Exclusion Criteria

Articles were selected for inclusion in this systematic review based on study type, participants, and interventions, as follows. Only randomized controlled trials, observational cohort studies, and case reports were included. For randomized trials, the researcher assessed the methodological approach to determine whether each study was based on a clearly described randomization method. The participants in the studies included in this review were patients with clinically diagnosed liver diseases or signs or symptoms of drug-induced liver damage who were undergoing antifungal therapy or had a recent history of oral antifungal administration. We selected studies that evaluated pharmacological and non-pharmacological interventions to prevent and manage fungal infection in liver disease patients and included only studies that compared interventions with controls such as placebos, standard treatment, or no treatment were selected. We placed no publication date restrictions on the searches and included only peer-reviewed journal articles with full text and that were published in English. Articles were excluded if they did not meet the inclusion criteria, were not peer-reviewed, were published in websites ending with dot com, or described medications other than antifungals. The primary outcomes were liver enzyme levels and the secondary outcomes were abdominal ultrasound and liver biopsy findings.

Information Sources and Search

Articles included in this review were obtained from searches in English-based databases with high quality and peer-reviewed articles, namely PubMed, CINAHL, and EMBASE. The searches were guided by the Cochrane Handbook for Systematic Reviews of Interventions, Version 5.1.0. We used keywords and Boolean search strategies, with terms such as OR, AND, and NOT used to identify relevant articles. The search carried in PubMed was based on the following combination of keywords: (“antifungal” OR “ketoconazole” OR “fluconazole” OR “griseofulvin” OR “terbinafine”) AND (“acute liver disease” OR “acute liver failure” OR “acute liver damage”). Articles were filtered to include only full-text articles, articles published from the databases’ inception to 2021, articles published in English, and peer-reviewed articles.

Study Selection

The retrieved articles were manually screened to remove duplicates and to remove irrelevant articles. The full texts of the remaining articles were then read to determine eligibility. We also assessed the bibliographies of the included articles for additional eligible studies not identified by the initial search, using the same eligibility criteria. The excluded studies were documented along with the reasons for exclusion. The final list of selected studies is shown in Table 1.

**Table 1:** Study ratings using Buckley's criteria Abbreviations: Y: Yes; N: No; U: Unclear

Study	Clear research question	Subject group appro­priate for study	Reliable and valid methods	Complete-ness of data	Controlled for confound-ers if non-RCT design	Statistical methods appropriate	Data justifies conclusion drawn	Study could be replicated	Prosp-ective study	Relevant ethical issues address-ed	Triangul-ation of data	Total score
García Rodríguez et al., 1999 [32]	Y	Y	Y	Y/U	U	Y	Y	N	Y	N	U	6
Perveze et al., 2006 [33]	Y	Y	Y	Y/U	U	Y	Y	N	N	N/U	U	6
Paredes et al., 2007 [34]	U	Y	Y	Y/U	U	Y/U	Y	N	N	U	U	5
Kao et al., 2013 [35]	Y	Y	Y	U	U/N	Y	Y	N	Y	U	U	6
Chan et al., 2014 [36]	U	Y	Y	U	U/N	Y	Y	N	N	N	U	5
Gayam et al., 2018 [37]	Y	Y	Y	U	Y	Y	Y	N	N	N	U	6

Data Collection Process and Data Items

We used a predefined datasheet to extract data from the selected articles, including the year of publication, author(s), article title, journal, country, setting, methods, participants, interventions, and outcomes.

Quality Assessment of Included Studies

The quality of all included studies was assessed using Buckley’s criteria to estimate the risk of bias in areas such as randomization, outcome measurement, selection of the reported result, missing outcome data, and overall bias. Studies were then categorized into one of three categories: low risk of bias, high risk of bias, and unclear risk of bias (some concerns) [31]. For the non-randomized studies, the assessed areas included the selection of participants into the study, classification of interventions, study intervention, outcome measurement, and selection of results.

## Review

Findings

Literature Search and Screening of the Selected Studies

**Figure 1:**
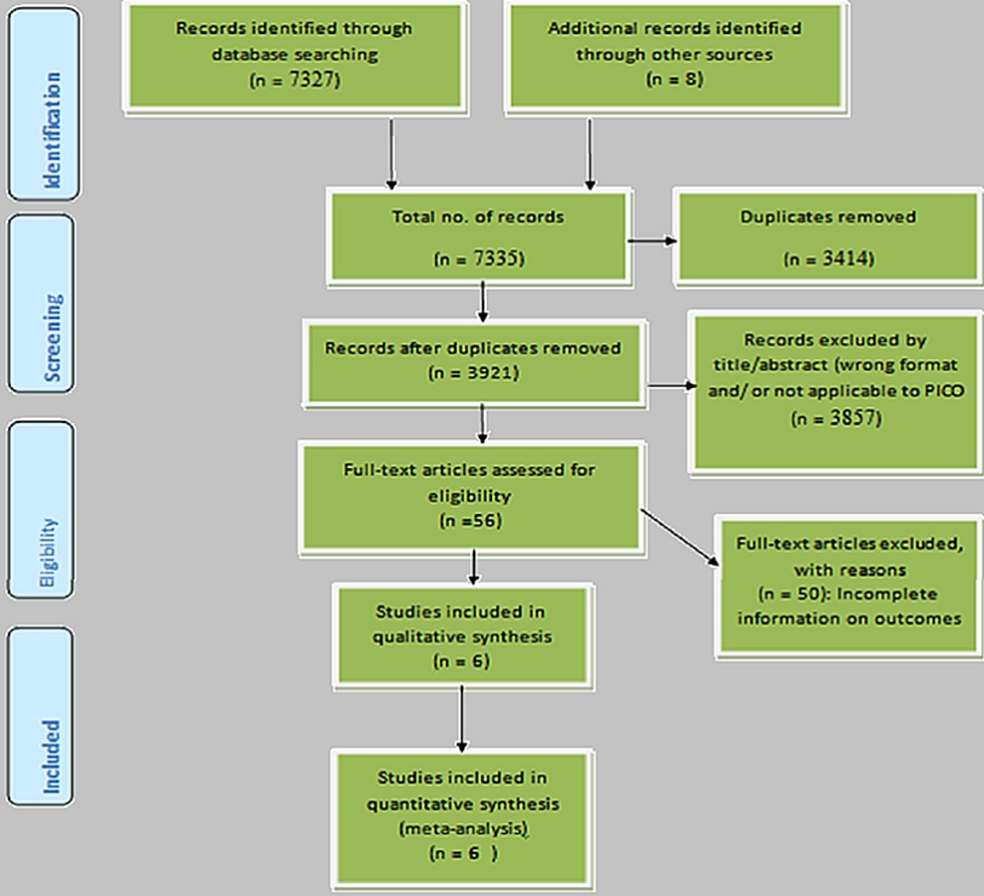
Preferred reporting items for systematic reviews and meta-analyses

The literature searches led to the retrieval of 7,335 articles, comprising 6,410 articles from PubMed, 652 from CINAHL, 265 from EMBASE, and eight additional studies from the bibliographies of selected studies. A flow chart of the screening process is shown in Figure 1. A total of 3,414 duplicates were identified and removed. The remaining 3,921 studies were then assessed for eligibility. First, we assessed the abstracts and titles to determine whether the objectives and the findings were relevant to the study, leading to the exclusion of 3,857 articles. The remaining 64 studies were subjected to full-text analysis. Following full-text analysis, 56 articles were excluded for the following reasons: 17 articles were not randomized control trials, observational cohort studies, or case reports, 21 articles had findings not specific to drug-induced liver damage, 14 articles were not yet published, and six did not clearly define both the sample and exposure. The remaining six articles were included in the systematic qualitative review.

Characteristics and Findings of Included Studies

The detailed baseline characteristics of all included studies are presented in Table 2. The total number of patients from all studies was 160680; most of these patients were from two cohort population studies. Rodríguez et al. evaluated the effect of terbinafine, itraconazole, and ketoconazole in patients aged 20-79 years and found that patients taking ketoconazole were more prone to acute liver damage than those taking terbinafine or itraconazole [32].

The study by Perveze et al. reported a case of terbinafine-induced severe liver failure requiring liver transplantation [33]. The patient was 50 years old with normal liver function tests during initial therapy of oral antifungal terbinafine for onychomycosis. After starting a three-month course of terbinafine, the patient started to notice dark urine and alcoholic stools and became progressively jaundiced. When he presented to an outpatient clinic, his liver enzyme levels were mildly elevated, so he was asked to discontinue terbinafine and was discharged. However, his condition worsened gradually, and when he visited the outpatient clinic again his serum enzyme levels were very high and the histological examination of his explanted liver revealed severe cholestasis, hepatocellular injury, and marked paucity of bile ducts [33].

A very similar case was reported by Paredes et al. In this case, the patient was a 57-year-old male with chronic hepatitis B virus (HBV) and normal liver enzymes who began oral terbinafine 250 mg once daily for 12 weeks to treat dermatophyte toenail onychomycosis. He developed jaundice and ascites, and three weeks after terbinafine was completed, his peak levels of aspartate aminotransferase and alanine aminotransferases were very high and a slight increase in bilirubin levels was noted; his platelet level had decreased notably. Transient autoantibodies and liver biopsy were consistent with acute autoimmune drug injury. The patient’s liver function studies began to normalize six weeks after discontinuation of terbinafine and the introduction of supportive care [34].

Kao et al. conducted a cohort study in 2013 which included more than 90,000 patients taking different antifungals including ketoconazole, fluconazole, itraconazole, terbinafine, and griseofulvin. Within this cohort, 58 cases of DILI were identified; of these cases, 12 patients were using fluconazole, 28 patients were using ketoconazole, two cases were using terbinafine, eight were using griseofulvin, and three were using itraconazole. The authors concluded that oral antifungal agents are associated with a low incidence of acute liver injury and that it may be fatal, especially for the elderly. They also found that a longer treatment duration may increase the risk of antifungal agent-induced liver injury, especially for ketoconazole [35].

The study by Chan et al. was a case report of a 34-year-old patient with Marfan syndrome and impaired kidney function undergoing intravenous fluconazole therapy, whose liver enzymes began to increase over the first few days of treatment. On day 6, the patient developed nausea and vomiting, and marked elevation of all serum enzymes was found. Serum enzyme levels and prothrombin time returned to their normal levels two weeks after discontinuation of the antifungal therapy [36].

Finally, a case study by Gayam et al. reported a rare case of dose-dependent acute liver injury, though this patient used to drink alcohol heavily. The patient was treated with fluconazole for inguinal candidiasis. His serum enzyme levels were normal on admission but drastically increased on the third day, and the patient developed acute liver failure. The drug-induced liver damage was classified as hepatocellular as the alanine transaminase /alkaline phosphatase ratio was >5. The patient’s condition improved three days after discontinuation of fluconazole, leading to a diagnosis of fluconazole‑mediated acute liver failure [37].

**Table 2:** Baseline characteristics and details of included studies ALT: Alanine Transaminase; ALP: Alkaline Phosphatase; AST: Aspartate Transaminase; DILI: Drug-Induced Liver Injury; GGT: Gamma-Glutamyl Transferase; IU/L: International Unit per Liter; IV: Intravenous; K: per 1,000 persons; mg/dL: Milligrams (mg) per Deciliter; mg: Milligram; mm3: Cubic Millimeter; μmol/L: Micromoles per Liter; UK: United Kingdom; US: United States of America; U/L: Units/Liter

Study ID/Year	Study design	Country	Sample size(n)	Exposure	Outcome
Rodrıguez et al., 1999 [32]	Cohort study	UK	69,830	Oral antifungals with a background rate of 0.6 per100 000 person-month	Ketoconazole and itraconazole were the two oral antifungals associated with a marked increase of clinical acute liver injury.
Perveze et al., 2006 [33]	Case study	US	1	Oral terbinafine for three months	terbinafine-induced severe liver a case of terbinafine-induced severe liver failure
Paredes et al., 2007 [34]	Case study	US	1	Oral terbinafine for twelve weeks	aspartate aminotransferase (1282 IU/L), alanine aminotransferase (1044 IU/L), and bilirubin (5.9 mg/dL); platelet level decreased to 77 × 103/mm3
Kao et al., 2013 [35]	Cohort study	Taiwan	90,847	Ketoconazole, 12 fluconazole, eight griseofulvin, three itraconazole and two terbinafine	The incidence rates (IR) of DILI per 10k persons 31.6, 4.9, 4.3, 3.6 and 1.6 for fluconazole, ketoconazole, griseofulvin, itraconazole and terbinafine, respectively.
Chan et al., 2014 [36]	Case study	Malaysia	1	34-year-old with elevated liver enzymes due to fluconazole (IV) 200 mg stat followed by IV 100 mg daily.	ALT and total bilirubin level further rose to 2394 U/L and 94 μmol/L on day 6
Gayam et al., 2018 [37]	Case study	US	1	IV fluconazole 200 mg daily	On day 3 AST of 25kIU/L, an ALT of 6.5k IU/L, a GGT of 210 IU/L, alkaline phosphatase of 130 IU/L, a total bilirubin 2.3 mg/ dL, and a direct bilirubin 0.4 mg/ dL

Discussion

Antifungal agents are considered to be a key cause of DILI. However, most studies in this area are case reports, and systematic analysis of this condition is extremely difficult. To our knowledge, there has been no systematic review in this field and topic. The present systematic review demonstrates the worldwide incidence rates of oral antifungal agent-induced liver injury. There was an increased risk of DILI in patients taking antifungal agents and the excess risk persisted after adjustment for age, gender, and comorbidities. Increased treatment duration with these drugs may increase the risk of liver injury and old age and fluconazole are risk factors for adverse liver outcomes. All six patients with fatal DILI had been administered fluconazole and were older than 60 years.

In a UK cohort treated with oral antifungal agents, the incidence rates of acute liver injury were 190 per 100,000 patients for ketoconazole, 10 for Iitraconazole, seven for terbinafine, and none for fluconazole or griseofulvin [32]. The DILI rates in the Taiwanese cohort study were even higher than this study with the highest DILI incidence rate found for fluconazole (316 per 100,000 persons) [35]. There was an increase in the incidence of DILI with increased duration of exposure to most antifungal agents; in the Taiwanese cohort study, the DILI incidence rate for ketoconazole increased from 49 per 100,000 patients in the overall cohort to 1,286 per 100,000 when the defined exposure duration was more than 30 days. Given the differences between these two studies, further prospective studies are needed to elucidate the impact of ethnic and genetic factors as well as the antifungal agent and exposure duration on the incidence and severity of DILI [35].

There is a very low frequency of oral terbinafine-induced severe liver damage. Previous systematic reviews of oral terbinafine have mostly discussed its cost and efficacy. One RCT reported a change in liver enzyme levels with terbinafine treatment, but follow-up after early withdrawal of the drug showed no further development of liver damage [38]. To obtain information on adverse reactions for this review, it is necessary to search for specific test results, such as liver enzyme levels and liver function abnormalities. Most terbinafine-induced liver injury cases recovered after discontinuation of the drug, and/or medical treatment. We found no reports of serious chronic liver damage. Only two patients required liver transplantation, and of these one had been exposed to terbinafine for three months and the other was taking terbinafine in combination with two other medications [39].

Clinicians should be aware that patients treated with antifungal agents for invasive fungal infections may have hepatic damage of differing degrees and origins. This complicates management and affects the efficacy and safety of antifungal therapy. Firstly, the metabolism and elimination of many antifungals are significantly altered by hepatic dysfunction, while DDIs are somewhat unpredictable compared to individuals with intact liver function. Moreover, it may be difficult to attribute further deterioration of liver biochemistry or function only to antifungals in patients with severe comorbidities and concomitant administration of other hepatotoxic drugs. In addition, precise estimates of hepatic function are currently unavailable. The Child-Pugh system, on which most dosage modifications in hepatic impairment are based, was initially developed to assess the prognosis of chronic liver disease and not the degree of hepatic dysfunction [14].

Although antimicrobials are the class of drugs that most commonly cause liver damage, acute liver failure is a rare outcome of antifungal use, with anecdotal prevalence mentioned in the literature. We scrutinized two studies describing acute liver injury rates in oral antifungal use and described the findings pertinent to our case. The first cohort analyzed 90,000 patients without any history of chronic liver damage who were taking oral antifungals and found a collective incidence of severe liver disease of three per 10,000 patients [35]. The second study analyzed almost 70,000 patients taking oral fluconazole and found an acute liver injury rate of 306 per 100,000 patients with six fatalities. Of note, all cases of fatal DILI in the Taiwanese cohort were older than 60 years, suggesting an association between age and poor prognosis [32].

This review draws attention to the discrepancies in the prevalence of oral antifungal agent-induced liver injuries. As some DILI cases present with asymptomatic transaminitis or elevated transaminase, physicians may simply examine or suspend oral antifungal agents without using liver injury codes. The main factor associated with fatal DILI in the studied articles was old age since most cases occurred in patients aged over 65 years. However, discovering whether factors are independently related to fatal DILI is complex; accurate interpretation of multivariate logistic regression requires reasonable event rates, possibly a minimum of ten events per variable [33].

## Conclusions

Antifungals are a well-known cause of elevated serum enzyme levels but have rarely been reported in the literature as agents associated with acute liver failure. Our review demonstrates that the risk of antifungal-induced acute liver failure is low but that it can be serious. Clinicians should be aware of this potential side effect and discontinue treatment promptly in cases of hepatic injury. Monitoring of liver function before and during treatment is strongly recommended.

## References

[1] Song JC, Deresinski S (2005). Hepatotoxicity of antifungal agents. Curr Opin Investig Drugs.

[2] Hay RJ (1993). Risk/benefit ratio of modern antifungal therapy: focus on hepatic reactions. J Am Acad Dermatol.

[3] Limper AH, Adenis A, Le T, Harrison TS (2017). Fungal infections in HIV/AIDS. Lancet Infect Dis.

[4] Kontoyiannis DP (2012). Invasive mycoses: strategies for effective management. Am J Med.

[5] Rodighiero V (1999). Effects of liver disease on pharmacokinetics. An update. Clin Pharmacokinet.

[6] Gupta NK, Lewis JH (2008). Review article: the use of potentially hepatotoxic drugs in patients with liver disease. Aliment Pharmacol Ther.

[7] Lewis JH, Stine JG (2013). Review article: prescribing medications in patients with cirrhosis - a practical guide. Aliment Pharmacol Ther.

[8] Palatini P, De Martin S (2016). Pharmacokinetic drug interactions in liver disease: an update. World J Gastroenterol.

[9] Pandit A, Sachdeva T, Bafna P (2012). Drug-induced hepatotoxicity: a review. J Appl Pharmaceut Sci.

[10] Lewis JH, Zimmerman HJ, Benson GD, Ishak KG (1984). Hepatic injury associated with ketoconazole therapy. Analysis of 33 cases. Gastroenterology.

[11] Lake-Bakaar DM, Abele G, Lindborg B, Soike KF, Datema R (1988). Pharmacokinetics and antiviral activity in simian varicella virus-infected monkeys of (R,S)-9-[4-hydroxy-2-(hydroxymethyl) butyl]guanine, an anti-varicella-zoster virus drug. Antimicrob Agents Chemother.

[12] Lee WM (2003). Drug-induced hepatotoxicity. N Engl J Med.

[13] Navarro VJ, Senior JR (2006). Drug-related hepatotoxicity. N Engl J Med.

[14] Bernal W, Wendon J (2013). Acute liver failure. N Engl J Med.

[15] Manning PR, Lee PV, Denson TA, Gilman NJ (1980). Determining educational needs in the physician's office. JAMA.

[16] Doß S, Potschka H, Doß F, Mitzner S, Sauer M (2017). Hepatotoxicity of antimycotics used for invasive fungal infections: in vitro results. Biomed Res Int.

[17] Collazos J, Mayo J, Martonez E, Diaz F (1995). Unusual liver toxicity due to the new antifungal agents fluconazole and itraconazole. Int Hepatol Commun.

[18] Reddy KR, Schiff ER (1995). Hepatotoxicity of antimicrobial, antifungal, and antiparasitic agents. Gastroenterol Clin North Am.

[19] Chien RN, Yang LJ, Lin PY, Liaw YF (1997). Hepatic injury during ketoconazole therapy in patients with onychomycosis: a controlled cohort study. Hepatology.

[20] Chiprut RO, Viteri A, Jamroz C, Dyck WP (1976). Intrahepatic cholestasis after griseofulvin administration. Gastroenterology.

[21] Ortiz-Quesada F, Méndez-Galván JF, Ritchie-Dunham J, Rosado-Muñoz FJ (1995). Organizational decision making in health: the case of dengue. (Article in Spanish). Salud Publica Mex.

[22] Lowe G, Green C, Jennings P (1993). Hepatitis associated with terbinafine treatment. BMJ.

[23] Gearhart MO (1994). Worsening of liver function with fluconazole and review of azole antifungal hepatotoxicity. Ann Pharmacother.

[24] Sakai MR, May ER, Imerman PM, Felz C, Day TA, Carlson SA, Noxon JO (2011). Terbinafine pharmacokinetics after single dose oral administration in the dog. Vet Dermatol.

[25] Tverdek FP, Kofteridis D, Kontoyiannis DP (2016). Antifungal agents and liver toxicity: a complex interaction. Expert Rev Anti Infect Ther.

[26] Piano S, Brocca A, Mareso S, Angeli P (2018). Infections complicating cirrhosis. Liver Int.

[27] Vilstrup H (2014). Hepatic encephalopathy in chronic liver disease: 2014 Practice Guideline by the American Association for the Study of Liver Diseases and the European Association for the Study of the Liver. Hepatology.

[28] Nahon P, Lescat M, Tran A (2017). Bacterial infection in compensated viral cirrhosis impairs 5-year survival (ANRS CO12 CirVir prospective cohort). Gut.

[29] Gadour E (2021). Acute on chronic liver failure (ACLF) during COVID-19; single UK based hospital experience. Gut.

[30] Pea F, Lewis RE (2018). Overview of antifungal dosing in invasive candidiasis. J Antimicrob Chemother.

[31] Buckley S, Coleman J, Davison I (2009). The educational effects of portfolios on undergraduate student learning: a Best Evidence Medical Education (BEME) systematic review. BEME Guide No. 11. Med Teach.

[32] García Rodríguez LA, Duque A, Castellsague J, Pérez-Gutthann S, Stricker BH (1999). A cohort study on the risk of acute liver injury among users of ketoconazole and other antifungal drugs. Br J Clin Pharmacol.

[33] Perveze Z, Johnson MW, Rubin RA (2007). Terbinafine-induced hepatic failure requiring liver transplantation. Liver Transpl.

[34] Paredes AH, Lewis JH (2007). Terbinafine-induced acute autoimmune hepatitis in the setting of hepatitis B virus infection. Ann Pharmacother.

[35] Kao WY, Su CW (2014). Risk of oral antifungal agent-induced liver injury in Taiwanese. Br J Clin Pharmacol.

[36] Chan JYM, Kiew CF, Chong CP (2014). Early onset hepatotoxicity associated with low dose fluconazole therapy in a critically ill patient. East J Med.

[37] Gayam V, Khalid M, Dahal S, Garlapati P, Gill A (2018). Hyperacute liver injury following intravenous fluconazole: a rare case of dose-independent hepatotoxicity. J Family Med Prim Care.

[38] Tausch I, Brautigam M, Weidinger G, Jones TC, The Lagos V Study Group (2008). Evaluation of 6 weeks treatment of terbinafine in tinea unguium in a double-blind trial comparing 6 and 12 weeks therapy. Br J Dermatol.

[39] Darkes MJ, Scott LJ, Goa KL (2003). Terbinafine: a review of its use in onychomycosis in adults. Am J Clin Dermatol.

